# A repeated measurement study investigating the impact of school outdoor environment upon physical activity across ages and seasons in Swedish second, fifth and eighth graders

**DOI:** 10.1186/1471-2458-14-803

**Published:** 2014-08-07

**Authors:** Peter Pagels, Anders Raustorp, Antonio Ponce De Leon, Fredrika Mårtensson, Maria Kylin, Cecilia Boldemann

**Affiliations:** Department of Sport Sciences, Linnaeus University, Kalmar, SE Sweden; Department of Public Health Sciences, Karolinska Institutet, Stockholm, SE Sweden; Department of Food and Nutrition, and Sport Sciences, University of Gothenburg, Gothenburg, SE Sweden; Swedish University of Agricultural Sciences, Alnarp, SE Sweden; Center for Epidemiology and Community Medicine, Stockholm County Council, Kragujevac, SE Sweden

**Keywords:** Outdoor environment, Physical activity, School yard, Accelerometers, Pupils

## Abstract

**Background:**

School children are confined to and exposed to outdoor environment that happens to be at their disposal during compulsory school time. The health-promoting potential of outdoor environment, and the use of it, is therefore important. We have studied the impact of school outdoor environment in terms of playground features, space, topography and vegetation upon the patterns of moderate to vigorous physical activity (MVPA) across ages and seasons in Swedish pupils at compulsory school.

**Methods:**

Four schools in the Middle and Southern parts of Sweden, with outdoor environments differing in playground features, space, topography and vegetation were analyzed during one school year. A sample of 196 children was drawn from eligible pupils in grades 2, 5 and 8, aged 7–14 years. PA was monitored with time-stamped Actigraph accelerometers GT3X+, measuring different intensity levels during outdoor time. Maps were used to mark places where the children stayed and what they did during outdoor time.

**Results:**

Mean MVPA during outdoor stay was 39 minutes for the entire school year, time in MVPA correlated positively with outdoor time, as did MVPA with used outdoor play area (p < 0.001). Outdoor MVPA declined with age, boys accumulated more MVPA than girls at all ages (p < 0.001). Ball play areas increased MVPA in 5^th^ graders in September and May (p < 0.001). Overall, ball play areas increased 5^th^ graders’ relative MVPA, and helped maintaining it with increasing age in boys but not in girls, whereas woodland stimulated and contributed to maintaining girls’ MVPA with increasing age. Outdoor temperature significantly impacted (p < 0.01) MVPA throughout all seasons.

**Conclusion:**

We conclude that school outdoor environment design and outdoor play time impact physical activity on a daily basis and may contribute to increasing girls’ physical activity and moderate the sharp decline in physical activity by age. The school outdoor environment may thus be a potential health promoter during school time.

## Background

From an early age physical activity (PA) promotes physical capacity, quality of life and self-esteem, as well as it reduces the risk of widespread diseases [1–3]. Conversely, physical inactivity has serious consequences, such as arterial stiffness [4], with signs of the metabolic syndrome occurring as early as in childhood and adolescence [5–7]. Childhood obesity for instance is most often sustained into adulthood with risks of developing e g. diabetes, cardiovascular diseases, and cancer [8–10]. Based on this knowledge there is evidence suggesting that children should be engaged in at least 60 minutes of moderate to vigorous physical activity (MVPA) on most days of the week to promote health benefits to be sustained into adulthood [1, 2, 11]. Far from all children reach these recommendations. Objective measurements of PA show prevalences of 60-80% for MVPA > 60 min a day in 9-10-year-old children [12, 13]. A clear drop in PA between 9 and 17 years will make healthy levels of physical activity even more remote [14].

Children between age six and 15 attending compulsory school are confined to and exposed to school environment that happens to be at their disposal when at school. For the sake of daily physical activity and its health benefit it is therefore important to design guidelines for healthy school environments that trigger physical activity, [15, 16]. Variables at both individual and school level have been shown to affect children's physical activity behavior [17, 18]. Thus the differences in children's physical activity, their individual characteristics and the school environment they use need to be better understood. The role of the outdoor environment may be a relevant predictor of PA, but the evidence of its actual role for PA is conflicting or unclear [15, 18–22]. It has been shown that preschool outdoor environment in terms of surface (vast, multileveled), and access to playable vegetation is associated with several health-promoting factors in children such as a leaner body, longer night sleep, and increased well-being [23]. Also, school children’s PA has been shown to be associated with the duration of outdoor stay and some environmental factors like playground area, play space (m^2^/child) and equipment availability [24–27]. Further, there is evidence that designed outdoor surfaces at primary school attract and thereby increase pupils’ PA during recess and outdoor play time [26–28].

Season has been stated to be an indispensable factor in analyzing behaviors surrounding PA [29]. Studies so far show conflicting results [30, 31]. Evidence also concludes that during school recess boys are physically more active than girls in almost all grades [14, 21], that PA decreases by age [32, 33], and that the outdoor environment impacts physical activity [34]. This study will further investigate the impact of outdoor environment at compulsory school upon the pupils’ patterns of physical activity considering season, age and gender.

## Methods

### Research design and participants

The present study is an integrated part of the KIDSCAPE II project that explores the co-beneficial impact of the outdoor environment on Swedish school children’s health at compulsory school. A repeated measurement study design was used to elucidate the characteristics of school environment that trigger spontaneous physical activity during fall, late winter, and late spring. The study was approved by the Regional Ethics Committee of Stockholm.

The sample was drawn from available 2^nd^, 5^th^ and 8^th^ graders (aged 7–14 years) at four municipal schools in mid-southern Sweden. The schools were selected considering differences in the overall layout of the outdoor environment, taking into consideration the total size of the school yard, its topography, surfaces with woodland, trees and bushes, and the presence of ball play areas, play equipment, and scheduled outdoor education. Further, the schools were selected to reflect the socio-economic composition of the municipalities of the whole country, with a majority of the population living in medium-sized cities outside or on the outskirts of a metropolitan area (Statistics Sweden). The ISCO code was applied for socioeconomic classification (European socio-economic classification, ISCO, 1988).

The parents of 259 pupils attending the selected schools were asked to let their children participate in the study. Both parents and their participating children signed a written consent to participate in the study. After obtaining permission, the school management, the pupils, teachers and parents received relevant and detailed information at an early stage and were given the opportunity to discuss the study. A sample of 196 (76%) agreed to participate.

### Procedures, data collection

In selecting school outdoor environments we considered all typical behavior settings and combinations of behavior settings that are representative for Northern and central European school outdoor environments. For a varied sample in terms of design and overall layout the school yards were selected in collaboration with a landscape researcher. To provide an estimate of the total school yard areas (m^2^), ball areas (soccer fields, basket fields, markings e.g.), green areas (woodland, grass, trees and bushes) and areas varying in use depending on season (used play area), the Google™ Earth Pro (GEP) software using aerial pictures of the playgrounds and the polygon measurement tool, described by Ridgers et al. 2010, were applied at each one of the four schools [26]. Topography (sum of hills and slopes), vegetation (amount of trees, bushes and grass), fixed equipment (swings, slides, climbing frames) and playground markings were inspected and counted by ocular inspection. The computed amount of space that the pupils used during the various seasons was based on the maps on which all the pupil’s positions during recess were marked. This patterned use of space was made up by different behavior settings (hereafter “used play space”). The percentage of used play space was obtained by dividing used play space by available school yard area. To calculate the variations between school, grade and seasons of the average play space (m^2^) used per child, the measured used play spaces were divided by the number of pupils for each grade.

Weather conditions were observed and ranked at AM and PM every day of fieldwork according to the following weather index, 1 = clear sky, 2 = partly cloudy, 3 = white cloudiness, 4 = grey cloudiness, 5 = precipitation. Temperature recordings were obtained from the Swedish Metrological and Hydrological Institute (SMHI).

Prior to data collection a questionnaire was filled in by the parents of the 2^nd^ graders and by the 5^th^ and 8^th^ graders themselves about family conditions (members of adults in the household, parents’ education and occupation, immigrant and/or indigenous, diseases etc.).

Data collection was carried out during five consecutive school days on three occasions during one school year, fall-September 2012, late winter-March 2013, and spring-May 2013. During each measurement period the pupils filled in a diary about leisure-time PA, considering other possible confounders (e.g. feeling unwell, sports etc. outside school). The pupils were weighed (scale: Beurer GS 27 Happystripes, CE Utrecht) and waists and heights measured using a measuring tape and a stadiometer (Seca 217, UK Birmingham) (Table 1).

**Table 1:** **Descriptive data of the participants by school, grade and sex**

										All schools			
		Art grass 1		Forest 2		City 3		Hill 4		Boys		Girls	
		Mean	SD	Mean	SD	Mean	SD	Mean	SD	Mean	SD	Mean	SD
2:nd grade (n = 74)	Height (cm)	136,5	± 7,5	131,6	± 7,8	132,7	± 4,8	135,2	± 5,7	133,9	± 6,7	133,6	± 6,6
	Weight (kg)	32,8	± 6,7	31,6	± 6,2	29,0	± 4,2	30,9	± 3,9	31,5	± 6,3	30,0	± 4,0
	Waist (cm)	65,2	± 8,3	64,6	± 6,4	61,0	± 3,8	60,2	± 5,6	63,4	± 7,0	61,6	± 5,0
	BMI (kg/m2)	17,5	± 2,1	18,1	± 2,1	16,4	± 1,6	16,9	± 1,5	17,4	± 2,2	16,8	± 1,4
5:th grade (n = 85)	Height (cm)	153,3	± 7,1	151,0	± 7,6	155,6	± 9,8	150,1	± 6,7	151,8	± 6,4	152,9	± 9,4
	Weight (kg)	46,3	± 10,4	45,5	± 12,0	45,0	± 11,6	43,6	± 7,2	44,3	± 8,4	45,8	±12,2
	Waist (cm)	70,3	± 11,1	70,3	± 10,0	67,3	± 8,5	69,5	± 6,8	69,8	± 8,8	69,0	± 9,6
	BMI (kg/m2)	19,6	± 3,7	19,8	± 3,5	18,4	± 3,2	19,3	± 2,5	19,1	± 2,8	19,4	± 3,6
8:th grade (n = 25)	Height (cm)	167,4	± 4,7	169,2	± 7,8	171,0	± 5,5	187,0	± 10,5	175,5	± 8,6	165,7	± 4,9
	Weight (kg)	64,4	± 9,0	69,2	± 15,9	59,4	± 7,1	74,4	± 7,7	65,7	± 12,2	63,7	±11,0
	Waist (cm)	82,5	± 6,8	81,7	± 16,2	73,4	± 6,2	73,0	± 7,8	75,1	± 10,5	80,7	± 9,7
	BMI (kg/m2)	23,0	± 3,6	24,0	± 5,1	20,3	± 1,8	21,2	± 0,3	21,3	± 3,6	23,1	± 3,2

PA was measured using hip-mounted accelerometers (Actigraph GT3X+ Activity monitors, US Pensacola) which enable time-stamped analysis of duration, intensity and location of activity in terms of indoor or outdoor activity (in this case important for the elimination of indoor stay). The accelerometers were activated on the first day of fieldwork and analyzed after the five consecutive days of each data collection period. Epochs were set at 10 seconds for detailed PA data [35, 36]. We applied previously validated (Evenson et al., 2008) and recommended cut-points set to <17 counts/10s for sedentary, 17 – 382 counts/10s for light PA, 383 – 682 counts/10s for moderate PA and >682 counts/10s for vigorous PA to estimate time spent in sedentary, light-, moderate-, and vigorous-intense PA in pupils aged 7–14 years [37]. Additionally, the built-in light sensor (Actilux) of the accelerometers for the registration of ambient light supplemented the separation of outdoor from indoor time registered by ocular observation. The sensitivity of the light sensor is 74% and specificity 86% [38], and valid for separating outdoor time in free living children 3–5 years [39]. Ambient light data were sampled and stored to memory at a 1 Hz rate. The downloaded data file was converted into an accumulated *.agd format with epoch lengths of 10 seconds, and average lux values for each epoch. In the Actigraph GT3X+ manual recommended values for indoor light are 1 – 500 lux (1 Hz) and for outdoor light >100 lux (1Hz). The cut point for lux value explaining outdoor time was set to >130 lux for average data of 10 second epochs, after comparing with observed values for in- and outdoor data, which were fairly consistent with newly presented validations of the GT3X+ light sensor [38, 39]. The accelerometers, attached to elastic belts, were put on upon arrival and removed at departure from school. The observer, one for each class, made sure that the accelerometers by were correctly mounted, worn outside and tightly strapped to the clothes. Each observer tracked the pupils of the designated class, marking the locations and activities for girls and boys separately on the school yard during recess. Sufficient data were obtained for 189 (88 girls) pupils, after the first, for 184 (85 girls) pupils after the second, and for 180 (86 girls) pupils after the third and last measurement period.

### Evaluation and statistical analysis

Data were analyzed using SPSS for Windows (22.0). School day accelerometer counts were condensed into time spent in MPVA during outdoor stay (observed and measured outdoor time) and divided by total outdoor time to provide percent of MPVA (%MVPA) of outdoor stay time. The mean numbers of measured schooldays per child were 4.3, 4.7 and 4.2 in September, March and in May respectively. A one-way ANOVA test showed no differences between measured days (1–5) and daily minutes in MVPA, p > 0.05, thus all pupils with at least one day of data were included in the analysis and the pupils’ average daily outdoor %MPVA used as the outcome variable. After scheduled lessons, the 2^nd^ graders spent the rest of the day at the after-school center situated at the school premises until their parents or older siblings fetched them. As differences in their MVPA between recess during the scheduled part of the day and outdoor stay at the after-school center were non-significant during all seasons (paired t-test, p > 0.05), and the children were confined to exactly the same outdoor environment, MVPA data were collapsed for the whole day.

Independent t- tests were applied to identify differences in physical activity means. Paired t-tests were used to evaluate the differences between seasons and outdoor physical activity. Intra-class correlation coefficients were calculated to estimate the influence from factors at school level upon %MVPA during outdoor stay. For bivariate analyses of confounder variables’ association vs. %MVPA the t-test, Spearman’s rho correlation coefficient and Pearson’s correlation coefficient were applied depending on which test was appropriate. Significantly associated variables were jointly tested in linear mixed model analysis, i.e. entered into the equation and sequentially removed (criterion for removal p ≥ 0.05) by highest p-value. Potential confounders related to PA and socio-economic status were non-significant and thus removed.

The choice of modeling was a linear regression model with random effects allowing for the clustering of measures within subjects, along seasons. With only three repeated measures within one school year, seasonality could play an important role in the autocorrelation patterns of the within-subject measures. A larger number of repeated measures per subject would have been necessary in order to correctly estimate the outcome pattern of autocorrelation. The matrix of variance-covariance for the correlated measures was therefore regarded as unstructured. The model specification regarded indicator variables for gender, for season when the measure was taken as well as for the school and grade the subject attended.

## Results

### Descriptive data

The schools were attended by 400–500 1-9^th^ graders, and located in cities with similar socio-economic status. Two of the schools, “Art Grass 1” and “Forest 2” were located in smaller municipalities (20156 and 26572 residents respectively) in eastern middle Sweden, both with woodland adjacent to the school yard. The other two schools, “City 3” and “Hill 4” were located in a medium-sized town (63700 residents) in southeast Sweden, the former bordering on the inner-city street scape of and the latter on the outskirts of town next to a block of flats. The school yard of “Art Grass 1” was flat and dominated by ball courts art grass with the surrounding woodland at some distance. “Forest 2” had paved areas around the building with two ball courts at one side of the building and a larger woodland area in its immediate surrounding with some play equipment and a small hill. The school yard of “City 3” was surrounded by a stone wall and dominated by paved surfaces containing ball courts of gravel, benches and manicured vegetation. The area of “Hill 4” with play equipment by the buildings sloped down from there and flattened into a plain towards the fence that surrounded the area (Table 2). The school areas of Art grass 1, Forest 2 and Hill 4 were >23 000 m^2^, the area of City 3 was approximately 13 000 m^2^. At Art Grass 1 and Forest 2 pupils could extend their activities beyond the “formal” school yard during outdoor stay, whereas City 3 and Hill 4 were surrounded by streets, houses and traffic. The weather during fieldwork was generally sunny with clear or partly cloudy skies, except for one rainy day in September at Forest 2, and one windy day with snowfall in March at Art Grass 1. Temperatures were above zero Celsius except during March, with temperature below zero Celsius at Art Grass 1 and Forest 2.

**Table 2:** **School playground outdoor environment predictors (sum) and relative MVPA (mean): A. yearly predictors and B. seasonal predictors**

		Art grass 1	Forest 2	City 3	Hill 4
A. yearly data	School outdoor area (m2)	24890	23482	13273	26613
	Outer limit of surface used for play (m2) 2nd grade	13915	9068	6562	12942
	Outer limit of surface used for play (m2) 5th grade	16592	12358	10111	12942
	Ball play area (m2)	5850	2800	2290	3075
	Green area (m2)	1720	7500	2200	2700
	Fixed equipment (sum)	13	14	16	17
	Outdoor pedagogical approach	No	Yes	No	No
	Percent in MVPA of the outdoor time (mean)	54.8	52.0	42.4	42.7
B. seasonal data	Temperature (°C)	15,9/-3,2/15,3	14/-4,3/15,3	13,1/3,3/14,8	13,1/3,3/14,8
Sept/March/May	Weather conditions (index median)*	2/3/4	3/2/2	3/2/2	3/2/2
	Used play area (% of playground) 2nd grade	79/72/59	95/41/95	76/82/78	41/41/52
	Used play area (% of playground) 5th grade	36/51/42	97/60/93	59/64/40	17/26/23
	Outdoor stay time (min) 2nd grade	123/82/120	84/78/123	109/129/137	123/111/124
	Outdoor stay time (min) 5th grade	76/42/49	73/109/58	51/98/71	102/81/110
	Outdoor stay time (min) 8th grade	NA/6/14	24/11/46	22/28/11	30/11/28
	Percent in MVPA of the outdoor time (mean)	55.6/52.4/56.4	56.2/47.6/51.9	52.3/30.4/44.2	45.9/40.9/41.2

*Weather index: 1 = cloudless, 2 = partly cloudy, 3 = white cloudiness, 4 = grey cloudiness, 5 = precipitation.

Of the participants 37 (19 girls) attended Art Grass 1, 51 (25 girls) Forest 2, 59 (21 girls) City 3, and 42 (23 girls) Hill 4, 12% (6% girls), were overweight (2^nd^ grade 11%, 5^th^ grade 13% and 8^th^ grade 13%). A majority of the 2^nd^ graders reported engaged on average in 2 sports activities/week, soccer, swimming, horse riding forming the dominating leisure time PA, 9% lived in single-parent households, 8% had a native tongue other than Swedish. The 5^th^ graders engaged on average in 3.2 activities /week after school, predominantly soccer, swimming and dancing, 6% lived in single-parent households, 10% had another native than Swedish. The 8^th^ graders, engaging on average in 3.6 activities /week after school, preferred ball games and gyms, 7% lived in single parent households, 7% had another native tongue than Swedish. Neither single household nor another native tongue than Swedish was correlated to physical activity in bivariate analysis in any grade.

### PA during outdoor time

Time spent at school and time spent outdoors was the same as the accelerometer wear time. Time was extracted from crude data stored in the accelerometers’ memory, and outdoor time separated from indoor time by Actilux data as described in the methods section. The pupils wore the accelerometers during their entire scheduled time during the school day and as long as they stayed at the school premises (2^nd^ graders). Throughout the seasons and during all days the pupils engaged in activities that enabled measurement by accelerometry, except for one 40-minute swimming lesson for each one of the 2^nd^ grade classes at Art Grass 1 and City 3 in September, and at The Hill 4 in May.

The 2^nd^ graders’ spent on an average 370 min/day at school (±49 min, time at the after-school center at the school premises included), the 5^th^ graders 339 min/day (±29 min) and the 8^th^ graders 311 (±32 min). The 2^nd^ graders mean daily outdoor stay was 113 minutes (±36 min), and their mean relative PA 87% (±12%), that of the 5^th^ graders was 78 minutes (±33), mean relative PA 79% (±15%) and that of the 8^th^ graders 22 minutes (±21), mean relative PA 73% (±22%). Outdoor time and absolute outdoor time in MVPA were positively correlated (r = 0.711, p < 0.001), however, relative MVPA deceased as outdoor time increased(r = -0.276, p < 0.001). Among 2^nd^ graders 93.3% reached the recommended time of 60 minutes spent in MVPA during the school day, which in, 51% of the 5^th^ graders and 5% of the 8^th^ graders did (measured period mean value).

### Impact of schools, season, grade and gender on PA during outdoor time

Outdoor activity was higher in September and May than in March. Art Grass 1 and Forest 2 were stronger predictors for time spent in MVPA during outdoor stay compared to City 3 and Hill 4, (Table 3). Boys spent more of their outdoor time in MVPA than girls, and 2^nd^ graders were more physically active than 5^th^ and 8^th^ graders during outdoor stay (Table 3).

**Table 3:** **Predictors for physical activity level during school outdoor stay (% of PA minutes)**

Mixed model, Typ III tests of fixed effects, repeated for seasons, F-values (Standard error)		Moderate to vigorous PA	
		%	SE
Season	**Intercept**	**46,43**	(1.62)
	Sept (n = 189)	(±) 0	
	March (n = 184)	(-) 10.99*	(1.48)
	May (n = 180)	(-) 4.55*	(1.48)
School			
	City 3 (n = 59)	(±) 0	
	Hill 4 (n = 42)	(+) 1.82	(1.72)
	Forest 2 (n = 51)	(+)10.76*	(1.62)
	Art Grass 1 (n = 37)	(+)12.94*	(1.78)
Grade			
	2:nd (n = 75)	(±) 0	
	5:th (n = 88)	(-) 6.17*	(1.32)
	8:th (n = 26)	(-) 4.97*	(1.93)
Gender			
	Girls (n = 88)	(±) 0	
	Boys (n = 101)	(+) 7.35*	(1.24)

The factors influencing time spent in different physical activity levels during pupils’ outdoor stay time, four Swedish schools 2012–13, analyzed by Mixed model. The direction of each relation is indicated, positive (+) and negative (-). Significant level between groups set to *p < 0.001.

Of all environmental factors only outdoor temperature had a significant impact (p < 0.01) on MVPA throughout all seasons (Spearman correlation coefficients, p < 0.01), and it was only in March that all of the outdoor settings (playground size, used play area, ball play area, and green play area) influenced MVPA in either direction in all 2^nd^ and 5^th^ graders. The used play area expanded along with increasing MVPA among all pupils (p < 0.01) except in 2^nd^ grade girls whose MVPA decreased (<0.01). On the other hand, green play areas increased MVPA significantly (p < 0.01) in this very group in both September and March (p < 0.01). Among 8^th^ graders no significant impact of any of the environmental factors on MVPA was observed.

**Figure 1:**
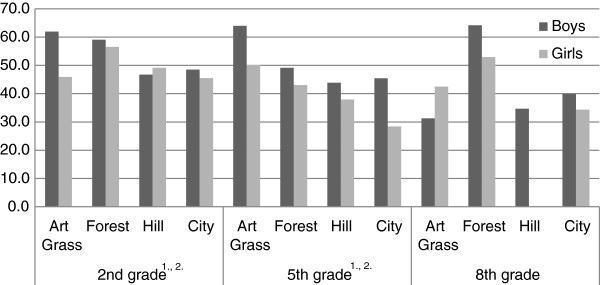
**Percent of outdoor time in MVPA in boys and girls, per schools and grades.** ^1.^Significant differences between girls at different schools, p<0.05, ^2.^Significant differences between boys at different schools, p<0.05.

MVPA among 2^nd^ grade girls at Forest 2 was significantly higher vs. all the other schools, as well as the difference between boys and girls was non-significant. Nor did boys’ and girls’ MVPA differ at Hill 3 and City 4, but the overall levels were significantly lower. Relative MVPA, though high at Art Grass 1, differed significantly between genders (Figure 1). MVPA among 5^th^ graders at Art Grass 1 and Forest 2, was significantly higher compared to the two other schools. At Art Grass 1 relative MVPA was the same as that of the 2^nd^ graders, but dropped among 5^th^ graders at the other schools. The steepest decline in relative MVPA was observed among girls, particularly at City 4. Throughout the schools a significant gender difference prevailed which was pronounced at City 4 (Figure 1).

## Discussion

The aim of this study was to further investigate the association between outdoor environment and level of physical activity across seasons and ages (grades) in Swedish school children at compulsory school. Relative time in MVPA was observed to differ during outdoor stay depending on the overall layout. Large play fields (Art Grass1) and woodland (Forest 2) both demonstrated significantly higher%MVPA during outdoor time, (12.94% and 10.76% respectively) than “city 3” with the smallest playground area.

Our results show a steep decline in PA between the 2^nd^ and 8^th^ grade which corresponds to findings by Sherar et al., based on all-day accelerometer data. They conclude that children’s PA decreases between 8 and 13 years, both by chronological and biological age [40]. But we also revealed a steep decline between 2^nd^ and 5^th^ grade in MVPA which is not clearly explained in other studies, at least during school time. Further studies are warranted to explain the decline in PA from childhood to adolescence. Also, attention needs to be paid considering the location and design of play spaces and equipment at schools that allow pupils to use the whole school ground [28]. For instance, the 2^nd^ and 5^th^ graders had play settings designed for their age or settings otherwise suited for free-living physical activity by their classrooms, which the classrooms of the 8^th^ graders had not.

Participants reaching the recommended time of 60 minutes spent in MVPA during the school day were 93.3% in 2^nd^ graders, 51% in 5^th^ graders, and 5% in 8^th^ graders. Other studies have concluded that with a cut-off point of approximately 2000 counts per minutes the proportion of children (age ≤12 years) who fulfill the PA recommendations ranged from 36 to 87%, [41]. A factor that may interact on PA is that time spent at school and time spent outdoors may differ between grades [17]. The 2^nd^ graders spent the longest time at school as well as the longest time outdoors and also presented with more MVPA than the 5^th^ and 8^th^ graders. Considering the low percentage of 8^th^ graders meeting the recommended level additional opportunities to be physically active such as scheduled physical education at school seem crucial for this age group.

Seasonal differences regarding MVPA outdoors were observed. Pupils of all grades spent most minutes in MVPA during fall and spring, compared to late winter. However significant seasonal differences were observed only among 2^nd^ and 5^th^ graders, except for in 2^nd^ grade girls. The largest seasonal shifts were represented by 2^nd^ grade boys between winter and spring with a mean difference of 26 minutes in MVPA. Those findings correspond with reported results showing that children are less active in winter than during other seasons, generally in areas with cold and long winters [29, 42, 43].

Relative MVPA (%MVPA) showed the same pattern as described above. Environmental factors that impact seasonal variations for MVPA in school children during school outdoor stay seem unclear. Vegetation and woodland have previously been shown to increase physical activity among older preschoolers [44] and among school children [34], particularly among girls. This is concordant with our observations, showing that girls at Forest 2 spent significantly more outdoor time in MVPA than girls did at the other schools. Open spaces seem to contribute to maintaining MVPA among pupils as they grow older. Among girls, woodland seems to fulfill that function as well (Figure 1). In accordance with previous studies we observed significant and positive correlations between time spent in MVPA during outdoor stay and temperature in 2^nd^ and 5^th^ grade school children as well [45, 46]. The contradictory results of the correlation between time outdoors and time spent in MVPA and relative MVPA of outdoor time could be explained by outdoor education activities, which were carried out over longer time periods in the form of lessons which may have contributed to lower activity. In March a whole day was scheduled for outdoor education with a mean of 210 minutes outdoors (n = 25) and 66.4 minutes in MVPA, i.e. 31.6% of the outdoor time which compared to the rest of the week was 6% lower. In addition the temperature in March dropped lower at Forest 2 than at any of the other sites (7.6 degrees lower than at City 3 and Hill 4), yet MVPA was high at Forest 2. Outdoor education during the cold part of the year may thus be important to increase time spent in MVPA.

The strength of this study was the use of objective monitoring both of outdoor time and of the intensity of PA. Further, comparing the Actilux readings to the recordings of outdoor times by direct observation made the data of clocked outdoor time more reliable. This is crucial as it is proven hard to recall details in physical activity patterns [17, 43]. Further, data were collected within a maximum time span of three weeks for each season. Though important for reliability this does not seem to be frequently applied in seasonal studies of physical activity, which is explained by expensive and time-consuming assessment. An important experience during fieldwork was the pupils’ occasional lack of adherence to the schedule for lessons and recess times which made direct observation necessary in order to obtain relevant and reliable data.

Weaknesses of the study were firstly the limited sample of schools. However, between the choice of investigating a more extensive sample combined with increased risk of unreliable fieldwork and/or superficial measurement (e.g. by proxy) or reliable but resource-consuming data collection in a smaller sample our choice was motivated by the variation in the outdoor school settings. We drew the conclusion that thorough fieldwork, though in a small sample, would deliver more valuable data, and – in spite of the few sites investigated – more generalizable data not least due to the variation of the outdoor school grounds. Secondly, the high drop-out among 8^th^ graders - possibly a consequence of unconsolidated identity protected by a high feeling of integrity - motivates caution in interpreting these data. The crude categorization of school ground quality and forms for tracking the children in this study also require qualitative studies. The understanding of how configurations of greenery, open spaces and play equipment contribute to physical activity for preschool children instead of only the presence of different elements [28] is still lacking for school children at compulsory school.

## Conclusions

Extended ball areas with art grass and green play areas with woodland seem to enhance MVPA in 2^nd^ and 5^th^ graders during outdoor stay. During late winter with the overall decline in activity outdoor education contributed to important activity among 5^th^ graders, even though the results also clearly indicate a relative decline in MVPA during this type of outdoor stay organized by adults. Our findings conclude that there is a stepwise decline in physical activity from the 2^nd^ to the 8^th^ grade and that the time spent outdoors during the school day could be part of the explanation. We also conclude that in all three grades boys are more physically active than girls which make it important to reveal what will improve girls’ physical activity in the school environment. However, woodland may contribute to maintaining levels of physical activity among girls. The school outdoor environment may thus be a potentially health promoting factor during compulsory school time.

### Ethical approval

Ethical approval for the study and its components was obtained from the Regional Ethics Committee of Stockholm, DNr.2011/370-31.

## Abbreviations

(PA): Physical Activity
(MVPA): Moderate to Vigorous Physical Activity
(GEP): Google™ Earth Pro
(SMHI): Swedish Metrological and Hydrological Institute.

## References

[1] Strong WB, Malina RM, Blimkie CJ, Daniels SR, Dishman RK, Gutin B, Hergenroeder AC, Must A, Nixon PA, Pivarnik JM, Rowland T, Trost S, Trudeau F (2005). Evidence based physical activity for school-age youth. J Pediatr.

[2] Janssen I, Leblanc AG (2010). Systematic review of the health benefits of physical activity and fitness in school-aged children and youth. Int J Behav Nutr Phys Activ.

[3] Martensson F, Boldemann C, Soderstrom M, Blennow M, Englund JE, Grahn P (2009). Outdoor environmental assessment of attention promoting settings for preschool children. Health Place.

[4] Sakuragi S, Abhayaratna K, Gravenmaker KJ, O'Reilly C, Srikusalanukul W, Budge MM, Telford RD, Abhayaratna WP (2009). Influence of adiposity and physical activity on arterial stiffness in healthy children: the lifestyle of our kids study. Hypertension.

[5] de Onis M, Blossner M, Borghi E (2010). Global prevalence and trends of overweight and obesity among preschool children. Am J Clin Nutr.

[6] Herouvi D, Karanasios E, Karayianni C, Karavanaki K (2013). Cardiovascular disease in childhood: the role of obesity. Eur J Pediatr.

[7] Weiss R, Dziura J, Burgert TS, Tamborlane WV, Taksali SE, Yeckel CW, Allen K, Lopes M, Savoye M, Morrison J, Sherwin RS, Caprio S (2004). Obesity and the metabolic syndrome in children and adolescents. N Engl J Med.

[8] Lemanne D, Cassileth B, Gubili J (2013). The role of physical activity in cancer prevention, treatment, recovery, and survivorship. Oncology.

[9] Aggoun Y (2007). Obesity, metabolic syndrome, and cardiovascular disease. Pediatr Res.

[10] Berenson GS, Srinivasan SR, Bao W, Newman WP, Tracy RE, Wattigney WA (1998). Association between multiple cardiovascular risk factors and atherosclerosis in children and young adults. The Bogalusa Heart Study. N Engl J Med.

[11] Blair SN, LaMonte MJ (2005). How much and what type of physical activity is enough? What physicians should tell their patients. Arch Intern Med.

[12] Owen CG, Nightingale CM, Rudnicka AR, Cook DG, Ekelund U, Whincup PH (2009). Ethnic and gender differences in physical activity levels among 9-10-year-old children of white European, South Asian and African-Caribbean origin: the Child Heart Health Study in England (CHASE Study). Int J Epidemiol.

[13] Steele RM, van Sluijs EM, Cassidy A, Griffin SJ, Ekelund U (2009). Targeting sedentary time or moderate- and vigorous-intensity activity: independent relations with adiposity in a population-based sample of 10-y-old British children. Am J Clin Nutr.

[14] Trost SG, Pate RR, Sallis JF, Freedson PS, Taylor WC, Dowda M, Sirard J (2002). Age and gender differences in objectively measured physical activity in youth. Med Sci Sports Exerc.

[15] Dessing D, Pierik FH, Sterkenburg RP, van Dommelen P, Maas J, de Vries SI (2013). School yard physical activity of 6–11 year old children assessed by GPS and accelerometry. Int J Behav Nutr Phys Activ.

[16] Griew P, Page A, Thomas S, Hillsdon M, Cooper AR (2010). The school effect on children’s school time physical activity: the PEACH Project. Prev Med.

[17] Sallis JF, Saelens BE (2000). Assessment of physical activity by self-report: status, limitations, and future directions. Res Q Exerc Sport.

[18] Ferreira I, van der Horst K, Wendel-Vos W, Kremers S, van Lenthe FJ, Brug J (2007). Environmental correlates of physical activity in youth - a review and update. Obes Rev.

[19] Baranowski T, Thompson WO, DuRant RH, Baranowski J, Puhl J (1993). Observations on physical activity in physical locations: age, gender, ethnicity, and month effects. Res Q Exerc Sport.

[20] Sallis JF, McKenzie TL, Alcaraz JE (1993). Habitual physical activity and health-related physical fitness in fourth-grade children. Am J Dis Child.

[21] Beighle A, Morgan CF, Le Masurier G, Pangrazi RP (2006). Children’s physical activity during recess and outside of school. J Sch Health.

[22] Raustorp A, Pagels P, Boldemann C, Cosco N, Soderstrom M, Martensson F (2012). Accelerometer measured level of physical activity indoors and outdoors during preschool time in Sweden and the United States. J Phys Act Health.

[23] Soderstrom M, Boldemann C, Sahlin U, Martensson F, Raustorp A, Blennow M (2013). The quality of the outdoor environment influences childrens health – a cross-sectional study of preschools. Acta Paediatr.

[24] Cardon G, Van Cauwenberghe E, Labarque V, Haerens L, De Bourdeaudhuij I (2008). The contribution of preschool playground factors in explaining children’s physical activity during recess. Int J Behav Nutr Phys Activ.

[25] Sallis JF, Conway TL, Prochaska JJ, McKenzie TL, Marshall SJ, Brown M (2001). The association of school environments with youth physical activity. Am J Public Health.

[26] Ridgers ND, Fairclough SJ, Stratton G (2010). Twelve-month effects of a playground intervention on children’s morning and lunchtime recess physical activity levels. J Phys Act Health.

[27] Haug E, Torsheim T, Sallis JF, Samdal O (2010). The characteristics of the outdoor school environment associated with physical activity. Health Educ Res.

[28] Mårtensson F, Jansson M, Johansson M, Raustorp A, Kylin M, Boldemann C (2014). The role of greenery for physical activity play at school grounds. Urban Forestry & Urban Greening.

[29] Tucker P, Gilliland J (2007). The effect of season and weather on physical activity: a systematic review. Public Health.

[30] Rowlands AV, Hughes DR (2006). Variability of physical activity patterns by type of day and season in 8-10-year-old boys. Res Q Exerc Sport.

[31] Rowlands AV, Pilgrim EL, Eston RG (2009). Seasonal changes in children’s physical activity: an examination of group changes, intra-individual variability and consistency in activity pattern across season. Ann Hum Biol.

[32] Tudor-Locke C, Lee SM, Morgan CF, Beighle A, Pangrazi RP (2006). Children’s pedometer-determined physical activity during the segmented school day. Med Sci Sports Exerc.

[33] Ridgers ND, Salmon J, Parrish AM, Stanley RM, Okely AD (2012). Physical activity during school recess: a systematic review. Am J Prev Med.

[34] Fjørtoft IK B, Sageie J (2009). Children in school yards: tracking movement patterns and physical activity in school yards using global positioning system and heart rate monitoring. Landscape and Urban Planning.

[35] McClain JJ, Abraham TL, Brusseau TA, Tudor-Locke C (2008). Epoch length and accelerometer outputs in children: comparison to direct observation. Med Sci Sports Exerc.

[36] Baquet G, Stratton G, Van Praagh E, Berthoin S (2007). Improving physical activity assessment in prepubertal children with high-frequency accelerometry monitoring: a methodological issue. Prev Med.

[37] Trost SG, Loprinzi PD, Moore R, Pfeiffer KA (2011). Comparison of accelerometer cut points for predicting activity intensity in youth. Med Sci Sports Exerc.

[38] Tandon PS, Saelens BE, Zhou C, Kerr J, Christakis DA (2013). Indoor versus outdoor time in preschoolers at child care. Am J Prev Med.

[39] Flynn JI, Coe DP, Larsen CA, Rider BC, Conger SA, Bassett DR (2014). Detecting indoor and outdoor environments using the ActiGraph GT3X+ light sensor in children. Med Sci Sports Exerc.

[40] Sherar LB, Esliger DW, Baxter-Jones AD, Tremblay MS (2007). Age and gender differences in youth physical activity: does physical maturity matter?. Med Sci Sports Exerc.

[41] Guinhouya BC, Samouda H, de Beaufort C (2013). Level of physical activity among children and adolescents in Europe: a review of physical activity assessed objectively by accelerometry. Public Health.

[42] Silva P, Santos R, Welk G, Mota J (2011). Seasonal Differences in Physical Activity and Sedentary Patterns: The Relevance of the PA Context. J Sports Sci Med.

[43] Kolle E, Steene-Johannessen J, Andersen LB, Anderssen SA (2009). Seasonal variation in objectively assessed physical activity among children and adolescents in Norway: a cross-sectional study. Int J Behav Nutr Phys Activ.

[44] Boldemann C, Blennow M, Dal H, Martensson F, Raustorp A, Yuen K, Wester U (2006). Impact of preschool environment upon children’s physical activity and sun exposure. Prev Med.

[45] Belanger M, Gray-Donald K, O'Loughlin J, Paradis G, Hanley J (2009). Influence of weather conditions and season on physical activity in adolescents. Ann Epidemiol.

[46] Feinglass J, Lee J, Semanik P, Song J, Dunlop D, Chang R (2011). The effects of daily weather on accelerometer-measured physical activity. J Phys Act Health.

[CR47] The pre-publication history for this paper can be accessed here:http://www.biomedcentral.com/1471-2458/14/803/prepub

